# Prenatal diagnosis of molybdenum cofactor deficiency

**DOI:** 10.1515/crpm-2026-0017

**Published:** 2026-09-30

**Authors:** Maria Teresa Araújo, Maria Vicente, Inês Carvalho, Ana Isabel Carocha, Carla Conceição, Ana Martins, Álvaro Cohen, Cláudia Rijo

**Affiliations:** Maternidade Dr. Alfredo da Costa, ULS São José, Lisbon, Portugal; Hospital Dona Estefânia, ULS São José, Lisbon, Portugal; Centro de Responsabilidade Integrado – Medicina e Cirurgia Fetal, ULS São José, Lisbon, Portugal

**Keywords:** prenatal diagnosis, molybdenum cofactor deficiency, ventriculomegaly, consanguinity

## Abstract

**Objectives:**

To describe the prenatal imaging findings and genetic diagnosis of molybdenum cofactor deficiency (MoCD) in two consecutive pregnancies, highlighting its variable prenatal presentation and diagnostic challenges.

**Case presentation:**

MoCD is a rare autosomal recessive metabolic disorder associated with severe neurological impairment and usually diagnosed postnatally. We report a consanguineous couple with two consecutive affected pregnancies. In the first, progressive ventriculomegaly and posterior fossa abnormalities were detected in the third trimester, raising suspicion of an underlying metabolic disorder, which was confirmed by genetic testing. In a subsequent monochorionic monoamniotic twin pregnancy, early imaging was unremarkable, but targeted genetic testing confirmed recurrence in both fetuses.

**Conclusions:**

MoCD may present prenatally with progressive central nervous system abnormalities. Combined imaging and molecular testing are essential for diagnosis, particularly in consanguineous couples, enabling timely counseling and reproductive decision-making.

## Introduction

Molybdenum cofactor deficiency (MoCD) is a rare autosomal recessive metabolic disorder resulting in functional loss of four molybdenum-dependent enzymes. Accumulation of toxic metabolites, including sulfite, S-sulfocysteine and thiosulfate, leads to severe neurological impairment [1], [2], [3].

Variants in *MOCS1*, *MOCS2* or *GPHN* disrupt MoCo biosynthesis, with *MOCS1* accounting for most cases [1], [2], [3].

The clinical manifestations of MoCD primarily affect the central nervous system and mimic hypoxic-ischemic encephalopathy [1], 2]. Most cases are diagnosed in the neonatal period, with refractory seizures, hypotonia, feeding difficulties and encephalopathy [1], 2].

Prenatal diagnosis is exceptionally rare and usually occurs in families with a previously affected child. We report progressive fetal neurosonographic abnormalities diagnosed as MoCD during pregnancy by genetic analysis, followed by recurrence in a subsequent pregnancy.

## Case presentation

The patient was a 31-year-old nulliparous woman from Pakistan, part of a consanguineous couple (first-degree cousins) with no significant medical history. The pregnancy was spontaneous and combined first-trimester screening – including maternal serum biochemistry (free β-hCG and PAPP-A) and ultrasound markers (nuchal translucency, ductus venosus Doppler, tricuspid flow, and assessment of the nasal bone) – indicated a low risk for common aneuploidies. However, the 22-week routine morphology scan raised suspicion of left ventriculomegaly, prompting referral for neurosonography. At 24 weeks, neurosonography confirmed isolated left ventriculomegaly, with the left lateral ventricle measuring 13.4 mm and no additional anomalies (Figure 1A).

**Figure 1:**
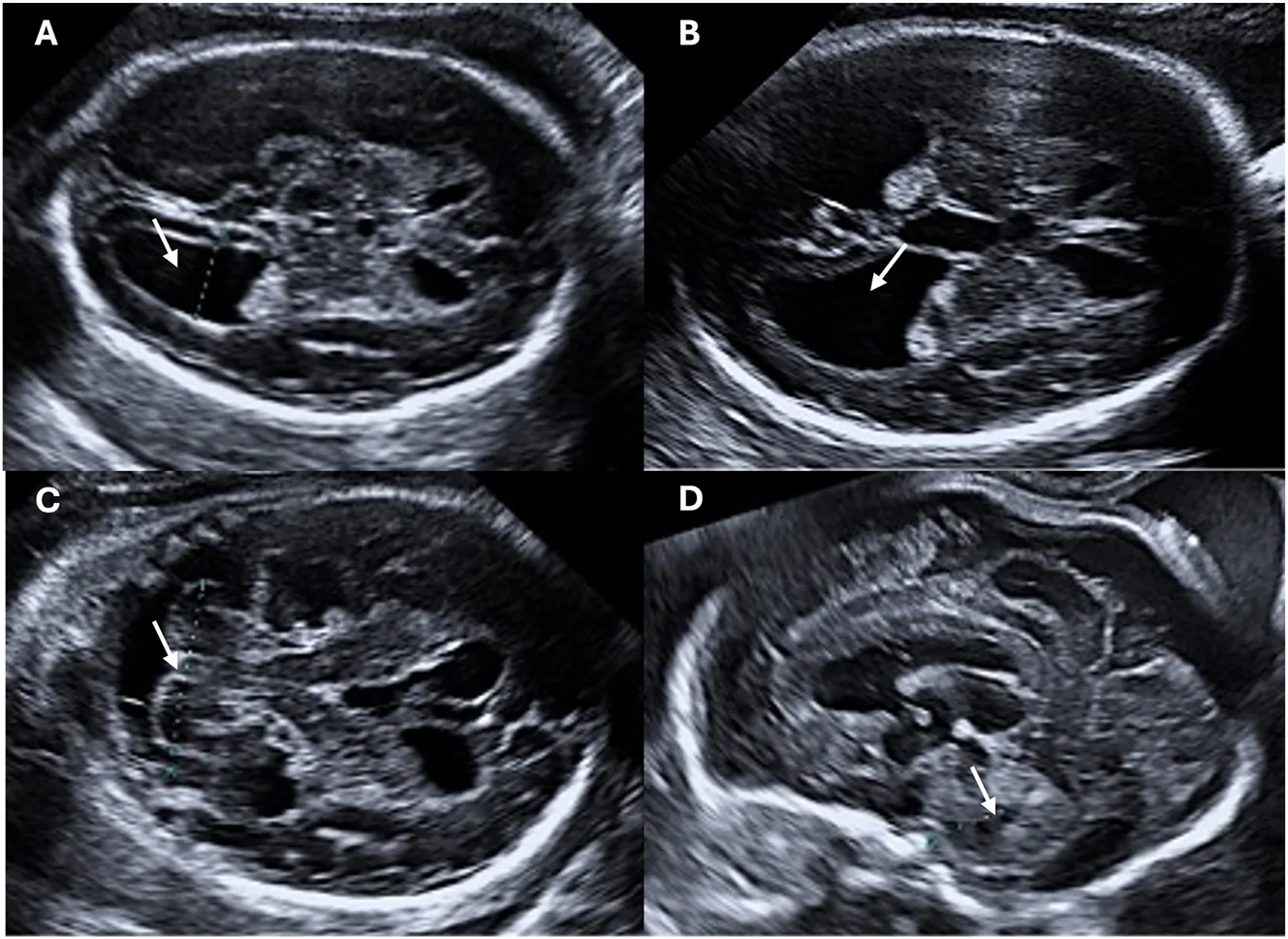
Prenatal imaging findings in molybdenum cofactor deficiency (MoCD) (A) Unilateral ventriculomegaly at 24 weeks. (B) Severe bilateral ventriculomegaly at 30 weeks. (C, D) Hydrocephalus, dilation of the third ventricle and cerebellum at the 10th percentile for growth, at 30 weeks.

Follow-up neurosonography at 26 and 28 weeks showed stable findings, with the left lateral ventricle measuring 13.0 mm and 13.5 mm, respectively, and no new structural abnormalities identified. However, at 30 weeks there was significant progression with severe bilateral ventriculomegaly (left ventricle measuring 20.2 mm and right ventricle 16.2 mm) (Figure 1-B), dilation of the third ventricle, and a cerebellum measuring at the 10th percentile (Figure 1C and D), reflecting progressive structural brain involvement. Fetal MRI was performed at 31 weeks to further characterize the central nervous system findings and exclude additional structural anomalies. This confirmed severe ventricular enlargement, demonstrated a retrocerebellar arachnoid cyst, small pons and cerebellum, and diffusely reduced cerebral parenchymal thickness.

Amniocentesis was performed, and initial investigations, including TORCH screening, quantitative fluorescence polymerase chain reaction (QF-PCR) for common aneuploidies (chromosomes 21, 13, 18, X and Y), and chromosomal microarray analysis, showed no abnormalities. Given the progression of cerebral abnormalities, further genetic testing was performed using trio exome sequencing (WES) at 30 weeks. This revealed a homozygous pathogenic variant [c.956G>A (p.(Arg319Gln)] in the *MOCS1* gene, confirming molybdenum cofactor deficiency. After genetic counseling, the couple decided to terminate the pregnancy. The fetal autopsy confirmed severe brain malformations with a smooth brain surface but no other visible dysmorphic features. Following termination of the pregnancy, the couple received genetic counseling regarding recurrence risk and future reproductive options, including preimplantation genetic diagnosis.

The patient returned eight months later with a spontaneous monochorionic–monoamniotic twin pregnancy. Combined first-trimester screening, including maternal serum biochemistry and first-trimester ultrasound markers, indicated a low risk for aneuploidy. Chorionic villus sampling (CVS) was offered; however, invasive testing was not performed at that stage, as the patient had not yet decided whether to proceed. At 18 weeks, she opted to undergo diagnostic testing, and amniocentesis was performed given the genetic diagnosis of the previous pregnancy. Both fetuses showed normal structural development with no significant weight discordance.

Targeted genetic analysis was performed specifically for the previously identified familial pathogenic variant [c.956G>A (p.(Arg319Gln)] in the *MOCS1* gene, given the history of severe fetal neurological abnormalities and the established association of this gene with a lethal autosomal recessive disorder. The analysis confirmed the presence of the same homozygous variant. Genetic counseling was provided, emphasising the poor fetal prognosis associated with this condition. After careful consideration, the couple decided to terminate the pregnancy.

## Discussion

Most published cases report MoCD complications in the neonatal period, as this is the most common clinical presentation. Prenatal diagnosis remains rare, with only a few documented cases. Table 1 summarizes published prenatal presentations of MoCD, including sonographic, MRI, and genetic findings.

**Table 1:** Summary of prenatal MoCD cases reported in the literature.

Author/year	Family history	Consanguinity	GA at prenatal findings	Prenatal findings	Genetic diagnosis	Gene/variant	Outcome	Notes
Carmi-Nawi et al., 2010	No prior history of affected child	Yes Second-degree cousins	35 weeks	Ventriculomegaly, corpus callosum dysgenesis, hypoplastic cerebellum, enlarged cisterna magna, multiple subcortical cystic	Postnatal genetic testing at 11 months	*MOCS1*, homozygous 251-418del	Neonatal seizures, feeding difficulties and global developmental delay	First case reported with prenatal CNS anomalies; Postnatal diagnosis based on genetic confirmation
Lubout et al., 2018	First case: Yes Second case: Yes	First case: Yes Second case: No	First case: 36 weeks Second case: 32 weeks	First case: ventricles appear slightly enlarged Second case: Mega cisterna magna	First case: Prenatal CVS Second case: Prenatal amniocentesis	First case: *MOCS1*, homozygous c.418+1G>A variation Second case: *MOCS1*, homozygous c.949C>T variation p.Arg317Cys		MRIs used to monitor disease progression; Prenatal suspicion and diagnosis based on family history
Alonzo Martínez et al., 2020	Yes	Yes	1st Pregnancy: No prenatal findings 2nd Pregnancy: 26 weeks	1st Pregnancy: No prenatal findings 2nd Pregnancy: enlarged cisterna magna	Postnatal genetic testing after 2nd fetal death	*MOCS1*, c.721delC/p.L241fs_246X	Neonatal death in both pregnancies	Prenatal findings only in 2nd pregnancy; Postnatal diagnosis based on genetic confirmation
Present case	No prior history of affected child	Yes	24 weeks	Bilateral ventriculomegaly, pontocerebellar hypoplasia, thin cerebral parenchyma, arachnoid cyst	Prenatal WES	*MOCS1*, homozygous c.956G>A (p.Arg319Gln)	Termination of pregnancy	First case with prenatal diagnosis confirmed by WES without previous affected sibling

The phenotype primarily affects the central nervous system, largely due to the accumulation of neurotoxic metabolites. Although the exact pathophysiology remains incompletely understood, it is believed that the neurological damage is primarily due to sulphite oxidase deficiency.

Some studies, including Reiss et al. (2005), suggest a maternal metabolic effect that delays the onset of the MoCD phenotype until birth through systemic clearance of sulfite during pregnancy [4]. Other studies suggest that placental clearance of toxic metabolites such as sulphite remains effective until late in pregnancy but may fail earlier in some cases [5]. This may explain the heterogeneity of prenatal findings, with morphologically normal fetuses in early pregnancy and the sudden onset of severe central nervous system anomalies in the third trimester, coinciding with the decline in maternal protection. The exact timing of this shift remains unclear, making it difficult to predict when sonographic signs of MoCD may appear. Our case closely supports this hypothesis, as neurosonographic findings remained relatively stable until 28 weeks before rapidly progressing between 28 and 30 weeks. This temporal pattern is consistent with a progressive loss of maternal metabolic protection during late gestation.

In 2010, Carmi-Nawi et al. published the first prenatal presentation of MoCD, characterised by multiple cerebral anomalies including ventriculomegaly, corpus callosum dysgenesis, hypoplastic cerebellum and enlarged cisterna magna, detected at 35 weeks’ gestation [6]. The infant subsequently developed seizures, feeding difficulties and global developmental delay. Based on these clinical manifestations and extensive laboratory and radiological studies, a diagnosis of MoCD was suggested at 11 months of age and later confirmed by genetic testing, which revealed homozygosity for the 251-418del mutation in the *MOCS1* gene.

In 2018, Lubout et al. described prenatal Magnetic Resonance Imaging (MRI) data from two cases of MoCD that showed signs of possible early brain damage. Both cases had a history of neonatal encephalopathy and death, with a subsequent diagnosis of MoCD. Given this history, both women underwent invasive testing (chorionic villus sampling and amniocentesis) during their next pregnancies, which confirmed variants in the *MOCS1* gene consistent with MoCD. Prenatal MRIs were performed to monitor the progression of MoCD and to detect early signs of brain damage. In the first case, signs of brain damage were observed at 32 weeks’ gestation and in the second at 36 weeks’ gestation.

More recently, in 2020, Alonzo Martínez et al. reported two consecutive pregnancies in which the first had no prenatal anomalies but the second had an enlarged cisterna magna at 26 weeks. In the first pregnancy, the newborn developed seizures shortly after birth and died within the first week. In the second pregnancy, prenatal imaging showed enlargement of the cisterna magna and a similar postnatal course followed. Following fetal necropsy and genetic testing, a diagnosis of MoCD was made.

Unlike the previously reported recurrent pregnancies, our second affected pregnancy involved a monochorionic monoamniotic twin gestation, introducing additional diagnostic considerations. Genetic discordance between monochorionic twins has been reported but remains exceptionally rare [7]. In this context, interpretation of the genetic findings was guided by the previously identified familial *MOCS1* pathogenic variant, the confirmed carrier status of both parents, and the very low likelihood of genetic discordance, allowing appropriate genetic counselling and pregnancy management.

MoCD may be considered in the differential diagnosis of progressive prenatal ventriculomegaly associated with cerebellar hypoplasia, especially in cases of consanguinity or a prior history of neonatal death with seizures [3]. Definitive diagnosis is based on the identification of a homozygous or a compound heterozygosity pathogenic variant in one of the genes involved in MoCo biosynthesis, most commonly *MOCS1* [8].

To our knowledge, this is the first reported prenatal diagnosis of molybdenum cofactor deficiency established by whole-exome sequencing in the absence of a previously affected sibling. Furthermore, the recurrence in a subsequent monochorionic monoamniotic twin pregnancy highlights the importance of early genetic counselling and targeted prenatal diagnosis in families at risk.
